# Subtomographic imaging of a polarisation sensitive phase pattern localised in phase space

**DOI:** 10.1038/s41598-024-52761-6

**Published:** 2024-02-01

**Authors:** Manpreet Kaur, Sheenam Saxena, Mandip Singh

**Affiliations:** Department of Physical Sciences, Indian Institute of Science Education and Research (IISER) Mohali, Sector-81, Mohali, 140306 India

**Keywords:** Optics and photonics, Physics, Optical physics

## Abstract

A transparent polarisation-sensitive phase pattern changes the phase of transmitted light without absorption, whereas this change of phase depends on the polarisation of incident light. A position-localised polarisation-dependent phase pattern is imprinted onto the phase-space of atoms by using atomic state dependent velocity-selective hole burning. A phase-space localised pattern is a higher dimensional generalisation of patterns localised in the position-space. Such a pattern cannot be imaged with a lens. The imprinted pattern is localised in a unique three-dimensional subspace of the six-dimensional phase-space of atoms. The phase-space localised pattern transforms the polarisation of light transmitting through it. This pattern is tomographically imaged at room temperature by measuring the intensity of the transmitted imaging laser beam of variable frequency with a camera after its polarisation analysis. Two sub-tomographs of the imprinted phase-space localised pattern are constructed. This paper presents a concept and experiment of imprinting and imaging of a polarisation-sensitive phase pattern localised in the phase-space.

## Introduction

**Figure 1:**
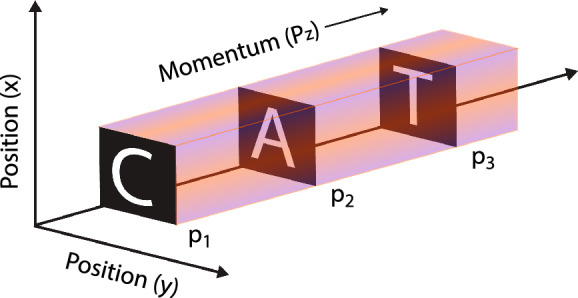
Three different light absorbing patterns, ${\textbf {C}}$, ${\textbf {A}}$ and ${\textbf {T}}$, which were initially localised in 2D position-space separately, are forming a single 3D localised pattern in the phase-space. This pattern is completely delocalised in the other 3D subspaces.

An experiment on the three-dimensional (3D) tomographic imaging of a pattern localised in the phase-space of atoms was introduced in Ref.^1^. The main motivation was to introduce the concept of a localised pattern and its tomographic imaging in a higher-dimensional space, such as a phase-space. A position-space object or pattern is defined as a function of position coordinates and time. A pattern is called stationary if it is independent of time. However, a phase-space object or pattern is defined as a function of position and corresponding momentum coordinates in phase-space by moving from 3D position-space to six-dimensional (6D) phase-space. In addition, if a phase-space pattern is independent of time, then it is called a stationary phase-space pattern. Thus, a 6D localised phase-space pattern is a function of three position and three momentum coordinates. Where momentum corresponds to the momentum of pattern constituents, which are atoms in the gaseous medium. A phase-space localised pattern cannot be imaged with a lens because a lens can only image position-localised objects. Therefore, phase-space localised patterns cannot be visualised with the eyes even when a pattern is emitting visible light. Human eyes and brain can interpret only those objects and patterns as visual objects that are defined in position-space. The concept of a pattern localised in the phase-space and its tomographic imaging was introduced through an experiment^1^. This experiment was performed by imprinting three different position-space localised patterns onto the phase-space of a Doppler broadened atomic gaseous medium by using velocity-selective hole burning^2–9^. In simple words, each position-space localised pattern is now placed in a unique 3D subspace of 6D phase-space comprising two position coordinates and one momentum coordinate. The resulting pattern corresponds to a single 3D phase-space pattern, which is delocalised in the 3D position-space consisting of orthogonal position coordinates *x*, *y* and *z* and in the 3D momentum-space consisting of corresponding momentum coordinates $p_{x}$, $p_{y}$ and $p_{z}$. Delocalisation implies that the different parts of different patterns, which are imprinted onto the phase-space, are overlapping with each other. Whereas a localised pattern is stationary without overlap. The imprinted pattern is only localised in a unique 3D subspace of the 6D phase-space of atoms, consisting of one momentum coordinate ($p_{z}$) and two transverse position coordinates (*x* and *y*) as shown in Fig. 1. Three different light absorbing two-dimensional (2D) objects in the form of the alphabets ${\textbf {C}}$, ${\textbf {A}}$ and ${\textbf {T}}$ represent three different position-space localised patterns, where a lighter region represents a complete transmission and a black region represents a complete absorption of light. Thus, the optical transmittance, which is only a function of position coordinates, represents a position-space localised pattern. These three position-localised patterns were imprinted onto the phase-space of atoms at room temperature. However, only in a subspace of 6D phase-space these three alphabets are localised at different momenta $p_{z}$ of atoms, *i.e.* ${\textbf {C}}$ at $p_{1}$, ${\textbf {A}}$ at $p_{2}$ and ${\textbf {T}}$ at $p_{3}$. These alphabets together represent a single phase-space object, which is now localised in a 3D subspace of 6D phase-space. This 3D localised phase-space object can be imaged by performing tomographic imaging in phase-space. As a result, all three alphabets can be selectively extracted from the phase-space to the position-space by the momentum selectivity of tomographic imaging, as demonstrated in Ref.^1^. A further tomography of a single alphabet localised in the phase-space is defined as sub-tomography.

Instead of momentum $p_{z}$, if the third coordinate is chosen to be the position *z* then, as experimentally shown in Ref.^1^, all the three alphabets overlap with each other in 3D position-space. There is no selectivity of different alphabets in the position-space because they are delocalised in a 3D position-space of atoms, and tomographic imaging cannot resolve them. Similarly, the imprinted pattern is delocalised in the 3D momentum-space. The imprinted 3D phase-space localised pattern was imaged by measuring the transverse intensity profile of the transmitted imaging laser beam with a camera. Which probes the depth of velocity-selective hole burning corresponding to different momentum components $p_{z}$ of atoms. Three different tomographs at three different momentum localisations were constructed, and a 3D tomographic image of the phase-space localised pattern was produced. The experiment is realised at wavelength 780 nm with 40 MHz frequency separation between the alphabets. From the application point of view, the atomic medium offers the ability to separate different images by varying the frequency of the imaging laser beam. Therefore, the phase-space localised pattern acts as an extremely narrow optical multi-band pass image filter with the band separation of a few tens of MHz at the optical frequency.

However, in the experiment described in Ref.^1^ one can imprint and image only the light-absorbing objects. An important question was raised after this paper. Imagine we have alphabets that are not light-absorbing but exhibit polarisation-dependent phase shift of the transmitted light. Therefore, these alphabets can modify the polarisation of the transmitted light without absorption. Can such different transparent patterns be imprinted as one object onto the phase-space of atoms. If it is possible, then how to extract tomographic position-space images from the imprinted phase-space localised pattern? This question is the motivation behind the concept and experiment presented in this paper. The present experiment is different from the experiment described in Ref.^1^ for the light-absorbing patterns. It is shown conceptually and experimentally that a position-localised polarisation-dependent phase shifting pattern, which is also known as a polarisation-sensitive phase pattern, can be imprinted as one pattern onto the phase-space of atoms. The imprinted phase-space localised pattern exhibits a shift in the polarisation-dependent phase of the transmitted imaging beam if it interacts with selected velocity classes of atoms, which can be used to extract tomographic images of the phase-space pattern.

In this paper, an experiment is presented to imprint a transparent polarisation-sensitive phase pattern onto the phase-space of an atomic gaseous medium. This pattern is localised in a unique 3D subspace of the 6D phase-space, consisting of two position coordinates (*x*, *y*) and one momentum coordinate $p_{z}$. The imprinted pattern is localised around $p_{z}=0$, and it is imaged corresponding to two different sections oriented perpendicular to $p_{z}$-axis around $p_{z}=0$. These two sectional images are considered as two sub-tomographs of the localised phase-space pattern, which itself is considered as a tomograph of finite width in contrast to the three different tomographs given in Ref.^1^. To do this experiment, a 2D position-localised transparent polarisation-sensitive phase pattern is produced with a spatial light modulator (SLM). The information of this pattern is transferred to an imprinting laser beam in the form of its transverse position-dependent polarisation. The imprinting laser beam interacts with a Doppler-broadened atomic gaseous medium. In contrast to the velocity-selective hole burning used in Ref.^1^, in this experiment, the imprinting laser beam produces an atomic state-dependent velocity-selective hole burning^10^ around $p_{z}=0$. Which is probed by a counter-propagating overlapping imaging laser beam to obtain two sub-tomographs at two different momentum locations around $p_{z}=0$ of the localised phase-space pattern.

## Transparent polarisation-sensitive phase pattern localised in the position-space

A transparent polarisation-sensitive phase pattern introduces a phase shift $\phi _{H}(x,z)$ in the horizontally ($\hat{z}$) polarised and $\phi _{V}(x,z)$ in the vertically ($\hat{x}$) polarised transmitted light at an arbitrary location (*x*, *z*) on the pattern. The transmitted electric field of a plane wave propagating along *y*-axis, for $\hat{z}$ polarised component, is $E_{H}e^{-i(2\pi \nu _{p} t-k y)} e^{i \phi _{H}{(x,z)}} \hat{z}$, where $E_{H}$ is the electric field amplitude, $\nu _{p}$ is frequency, $k=2\pi /\lambda$ is the magnitude of propagation vector at wavelength $\lambda$ and $\phi _{H}{(x,z)}$ is the phase shift introduced by the pattern. Similarly, the transmitted electric field for $\hat{x}$ polarised component is $E_{V}e^{-i \left( 2\pi \nu _{p} t-k y \right) } e^{i \phi _{V}{(x,z)}} \hat{x}$, where $\phi _{V}{(x,z)}$ is the phase shift introduced by the pattern and $E_{V}$ is the electric field amplitude. If the incident light is linearly polarised such that its plane of polarisation is oriented at an angle $-\,45^{\circ }$ *w.r.t* *z*-axis. Which is an equal superposition of horizontally and vertically polarised components, then the transmitted light exhibits a change in polarisation such that the transmitted electric field is $-E_{t}e^{-i \left( 2\pi \nu _{p} t-k y \right) } e^{i \phi _{V}{(x,z)}} \left( \hat{x} -e^{i\phi (x,z)}\hat{z}\right) /\sqrt{2}$, where $E_{t}$ is the electric field amplitude of the transmitted light. Therefore, a change in polarisation depends on the position-dependent phase difference given by $\phi (x,z)=\phi _{H}(x,z)-\phi _{V}(x,z)$, which is a representation of the birefringent property of the transparent polarisation-sensitive phase pattern localised in *x*-*z* position-space.

**Figure 2:**
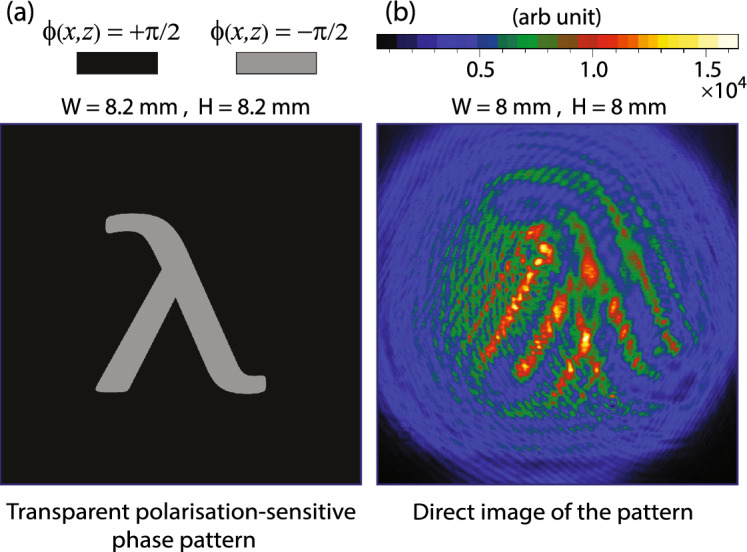
(**a**) A position-space localised transparent polarisation-sensitive phase pattern with phase shift $\phi (x,z)$, where the width (*W*) and height (*H*) of the pattern are both 8.2 mm. (**b**) Transverse intensity profile of the retro-reflected laser beam measured by a camera at a distance about 45 cm from the pattern without polarisation analyser.

**Figure 3:**
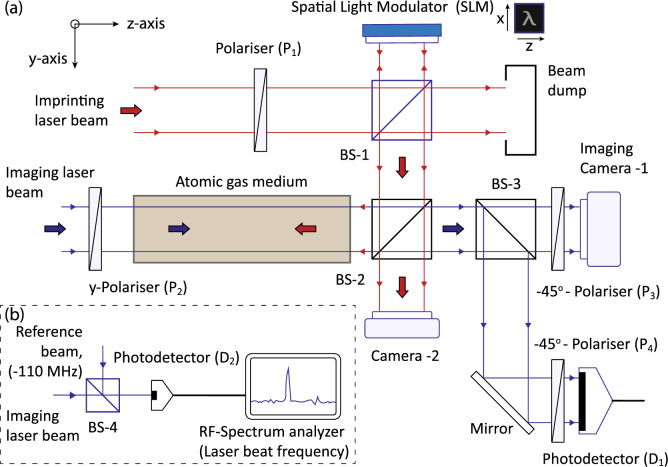
(**a**) A schematic diagram of the subtomographic imaging experiment in phase-space. (**b**) A part of the experiment to measure a frequency difference of lasers by time domain interference on a fast response photodetector. The vertical *x*-axis is perpendicular to the plane of page.

In Fig. 2a, a transparent polarisation-sensitive phase pattern is shown, where the darker region represents the phase difference, $\phi (x,z)=+\pi /2$ and the lighter region represents $\phi (x,z)=-\pi /2$. Therefore, transmitted light just close to the pattern is right circularly polarised corresponding to the darker region and left circularly polarised corresponding to the lighter region. If this transparent polarisation-sensitive phase pattern is imaged directly with a camera, then no intensity image is formed. An image captured by the camera, of the transverse intensity profile of transmitted light, is shown in Fig. 2b. The pattern is not formed; however, only the edges appear in the image due to diffraction at the boundaries, as the image is captured at a distance of 45 cm from the pattern. A polarisation-sensitive phase pattern can be imaged with polarisation contrast imaging if the pattern is localised in the position-space by passing the transmitted light through a polarisation analyser, whose output intensity depends on the polarisation of the incident light. Pure phase contrast imaging and microscopy are extensively explored fields of research^11–23^. In the context of quantum imaging, experiments with hyper-entangled photons have been reported to image transparent polarisation-sensitive phase patterns^24,25^. However, a phase-space localised transparent polarisation-sensitive phase pattern cannot be imaged with these techniques. In the experiment presented in this paper, a transparent polarisation-sensitive phase pattern is produced by a reflection-type SLM. A linearly polarised light with its plane of polarisation oriented at an angle $-\,45^{\circ }$ *w.r.t.* *z*-axis is incident perpendicular to the SLM surface. The retro-reflected light from SLM exhibits a position and polarisation-dependent phase shift. This configuration is equivalent to a transmission-type SLM, where a phase shift is imprinted on the transmitted light. The reflecting surface of the SLM is oriented perpendicular to the *y*-axis as shown in Fig. 3a. The SLM introduces a position-dependent phase shift only for the horizontal polarisation component of light, whereas the phase of the vertical polarisation component is uniformly shifted. This produces the required phase shift $\phi (x,z)$. A pattern shown in Fig. 2a is displayed on the SLM, and the phase shift information is transferred to the retro-reflected laser beam for imprinting onto the phase-space as described in the next section.

## Imprinting of a polarisation-sensitive phase pattern onto a 3D phase-space

To imprint a pattern onto the phase-space of a Doppler broadened atomic gaseous medium, the imprinting laser beam is passed through the atomic gaseous medium as shown in Fig. 3a. The imprinting laser beam is first incident on the SLM, and a retro-reflected beam is further reflected by a polarisation-independent beam splitter BS-2 into a glass cell filled with atomic gas. After a reflection by BS-2, the polarisation-sensitive phase shift is denoted by $\phi (x,y)$. Note that after this reflection, the direction of propagation of the imprinting laser beam is changed from the $+y$-axis to the $-z$-axis therefore, the variables of phase shift are changed. A transverse intensity profile of the imprinting laser beam of frequency $\nu _{p}$ propagating in the atomic gaseous medium along $-z$-axis is given by $I_{p}(x,y, \nu _{p})$, which is considered to be uniform. However, a transmitted part of the imprinting laser beam by BS-2 is imaged by a camera-2 without any lens, which captures a direct intensity image as shown in Fig. 2b of the position-space localised transparent polarisation-sensitive phase pattern displayed on the SLM as given in Fig. 2a. A polariser P_1_ is adjusted such that the plane of polarisation of light incident on the SLM is oriented at an angle $-\,45^{\circ }$ *w.r.t* the horizontal *z*-axis. The light transmitted by the beam splitter BS-1 is absorbed by a beam dumper to minimise scattered light falling on the camera.

A position-space localised pattern is imprinted onto the phase-space of atoms by using atomic state-dependent velocity-selective hole burning in the Doppler-broadened atomic gaseous medium. The polarisation of imprinting laser beam is transverse position dependent according to the pattern displayed on the SLM. Therefore, the electric field of the imprinting laser beam propagating in the atomic gaseous medium along $-z$-axis is given by

1 $$\begin{aligned} \vec {E}_{p}=E_{o}e^{-i(2\pi \nu _{p} t+k z)} \left( \frac{\hat{x} +e^{i\phi (x,y)}\hat{y}}{\sqrt{2}}\right) \end{aligned}$$

where $E_{o}$ is the amplitude of the electric field and $k=2\pi /\lambda$ is the propagation constant. Therefore, for some regions on the wavefront of the beam where $\phi (x,y)=\pi /2$, the imprinting laser beam is $\hat{\sigma }^{+}$ circularly polarised (left circular polarisation). For regions where $\phi (x,y)=-\pi /2$, the imprinting laser beam is $\hat{\sigma }^{-}$ circularly polarised (right circular polarisation). This $\sigma$-polarisation convention is defined *w.r.t.* the right-handed coordinate system given in Fig. 3. Note that after a reflection by BS-2 the right circular polarisation becomes the left circular polarisation and vice versa, but polarisation in the $\sigma$ convention remains unchanged. The position-dependent circular polarisation produces an atomic state-selective excitation of atoms. Consider a ground state of an atom $|F_{g}, m_{F}\rangle$ with energy $E_{g}$ and an excited state $|F_{e}, m'_{F}\rangle$ with energy $E_{e}$, where corresponding magnetic quantum states are labeled by $m_{F}$ and $m'_{F}$. Here *z*-axis is considered as a quantization axis. For a stationary atom, maximum absorption occurs when the frequency of light is $\nu _{o}=(E_{e}-E_{g})/h$, where *h* is the Planck’s constant. Regions on the wavefront of the imprinting laser beam, where the beam is $\hat{\sigma }^{+}$ polarised, light excites atoms from a ground state $|F_{g}, 0\rangle$ to an excited state $|F_{e}, +1\rangle$. Regions where the wavefront is $\hat{\sigma }^{-}$ polarised, light excites atoms from $|F_{g}, 0\rangle$ to $|F_{e}, -1\rangle$, considering a single magnetic sublevel of the ground state and three magnetic sublevels of the excited state. At room temperature, atoms are moving with a Maxwell velocity distribution. Therefore, atoms of a particular velocity class $v_{r}$ moving along the *z*-axis are in resonance with the imprinting laser beam of frequency $\nu _{p}$ because of the Doppler shift, which is given by $v_{r}=2\pi (\nu _{o}-\nu _{p})/k$, where a propagation constant *k* is defined in the rest frame of reference and relative to this frame the velocity is measured. Atoms moving along a transverse direction to the beam propagation experience a negligible influence of the transverse Doppler shift of light at room temperature. Consider, *N* is the number of atoms per unit volume at the absolute temperature *T*, a fraction of atoms in an interval $d v_{z}$ around *z*-component of velocity $v_{z}$ of atoms is given by the Maxwell velocity distribution, $f(v_{z}) dv_{z}= \left( m/2 \pi k_{B} T \right) ^{1/2}e^{-m v^{2}_{z}/2 k_{B} T} dv_{z}$, where $k_{B}$ is the Boltzmann constant and *m* is mass of an atom. The imprinting laser beam causes velocity-selective and magnetic state selective excitation of atoms. Therefore, for $\hat{\sigma }^{+}$ polarised regions, the atom number difference per unit volume of atoms in the ground state $|F_{g}, 0\rangle$ with atomic density $n_{1}(x,y,v_{z})$ and in the excited state $|F_{e}, +1\rangle$ with atomic density $n^{+}_{2}(x,y,v_{z})$ is given by

2 $$\begin{aligned} n_{1}(x,y,v_{z})-n_{2}^{+}(x,y,v_{z})=\frac{N f(v_{z})}{1+\frac{I^{+}_{p}({x,y,\nu _{p}})\Gamma ^{2}}{4I^{+}_{s}\left[ (2 \pi \nu _{p}-2 \pi \nu _{o}+kv_{z})^{2}+\frac{\Gamma ^{2}}{4}\right] }} \end{aligned}$$

where $I^{+}_{s}$ is the saturation intensity of $\sigma ^{+}$ atomic transition, $I^{+}_{p}({x,y,\nu _{p}})$ is the intensity of imprinting laser beam at $\hat{\sigma }^{+}$ polarised regions and $\Gamma$ is the linewidth of the transition. It is also assumed that the atomic population in the ground state is much larger than the excited state population. Similarly, for $\hat{\sigma }^{-}$ polarised regions, the atom number difference per unit volume of atoms in the ground state $|F_{g}, 0\rangle$ with atomic density $n_{1}(x,y,v_{z})$ and in the excited state $|F_{e}, -1\rangle$ with atomic density $n_{2}^{-}(x,y,v_{z})$ is given by

3 $$\begin{aligned} n_{1}(x,y,v_{z})-n_{2}^{-}(x,y,v_{z})=\frac{n f(v_{z})}{1+\frac{I^{-}_{p}({x,y,\nu _{p}})\Gamma ^{2}}{4I^{-}_{s}\left[ (2 \pi \nu _{p}-2 \pi \nu _{o}+kv_{z})^{2}+\frac{\Gamma ^{2}}{4}\right] }} \end{aligned}$$

where $I^{-}_{s}$ is the saturation intensity of $\sigma ^{-}$ atomic transition and $I^{-}_{p}({x,y,\nu _{p}})$ is the intensity of imprinting laser beam at $\hat{\sigma }^{-}$ polarised regions. For a uniform beam, $I^{+}_{p}({x,y,\nu _{p}})=I^{-}_{p}({x,y,\nu _{p}})= I_{p}({x,y,\nu _{p}})$. This magnetic state-dependent and velocity-selective atomic population difference given by $n_{1}(x,y,v_{z})-n_{2}^{+}(x,y,v_{z})$ and $n_{1}(x,y,v_{z})-n_{2}^{-}(x,y,v_{z})$ together represent a 3D phase-space localised pattern because it is independent of time, and it is defined in a 3D phase-space comprised of coordinates *x*, *y* and $p_{z}=mv_{z}$. The phase $\phi (x,y)$ information of a pattern localised in a position-space was carried by the imprinting laser beam field Eq. (1), which is transferred to atoms resulting a pattern localised in a 3D phase-space of atoms. In a 3D position-space, atoms are randomly moving and an excited atom can be anywhere on *z*-axis. Therefore, this pattern is delocalised in the 3D position-space along the *z*-axis. Since the atomic resonances can be probed with a high resolution therefore, the phase-space localised pattern can be tomographically imaged by varying the frequency of a very narrow linewidth probe laser.

## Imaging of a 3D phase-space localised pattern

To image a 3D phase-space localised pattern tomographically, a counter-propagating horizontally *y*-polarised imaging laser beam of uniform intensity $I_{r}(x,y,\delta \nu )$, frequency $\nu _{r}$ is overlapped with the imprinting laser beam and passed through the atomic gaseous medium as shown in Fig. 3a. A linearly polarised beam is a linear superposition of $\hat{\sigma }^{+}$ and $\hat{\sigma }^{-}$ circular polarisations. Therefore, each circular polarisation component of the imaging laser beam experiences a different absorption and refractive index at different transverse locations (*x*, *y*) and detuning $\delta \nu =\nu _{r}-\nu _{o}$, because the medium is excited magnetic state selectively and velocity-selectively by the imprinting laser beam. A particular location of a tomographic section on the momentum axis is $p_{z}=2 m\pi \delta \nu /k$, which is selected by the frequency detuning $\delta \nu$ of the imaging laser beam. The absorption coefficient $\alpha ^{+}(x,y,\delta \nu )$ for $\hat{\sigma }^{+}$ polarised component of the imaging laser beam is a convolution of the atomic population difference $(n_{1}(x,y,v_{z})-n_{2}^{+}(x,y,v_{z}))$, and the absorption cross-section for a corresponding transition of an atom, which is given by

4 $$\begin{aligned} \alpha ^{+}(x,y,\delta \nu ) =\int ^{\infty }_{-\infty } \big (n_{1}(x,y,v_{z})-n_{2}^{+}(x,y,v_{z})\big ) \frac{\sigma ^{+}_{o}(\frac{\Gamma ^{2}}{4})dv_{z}}{(2 \pi \delta \nu - kv_{z})^{2}+\frac{\Gamma ^{2}}{4}} \end{aligned}$$

where $\sigma _{o}^{+}$ is the peak absorption cross-section of the $\sigma ^{+}$ atomic transition. Similarly, the absorption coefficient $\alpha ^{-}(x,y,\delta \nu )$ of $\hat{\sigma }^{-}$ polarised component of the imaging laser beam is given by

5 $$\begin{aligned} \alpha ^{-}(x,y,\delta \nu ) =\int ^{\infty }_{-\infty } \big (n_{1}(x,y,v_{z})-n_{2}^{-}(x,y,v_{z})\big ) \frac{\sigma _{o}^{-}(\frac{\Gamma ^{2}}{4})dv_{z}}{(2 \pi \delta \nu - kv_{z})^{2}+\frac{\Gamma ^{2}}{4}} \end{aligned}$$

where $\sigma _{o}^{-}$ is the peak absorption cross-section of the $\sigma ^{-}$ atomic transition. The absorption coefficient is reduced due to the saturation of absorption, when imprinting and imaging laser beams interact with the same velocity class. A change in the absorption is different for $\hat{\sigma }^{+}$ and $\hat{\sigma }^{-}$ components of polarisation at different transverse locations in the atomic medium. As a consequence of Kramers-Kronig relations^10^, a change in the absorption leads to a change in the refractive index for each $\sigma$-polarisation component at different transverse locations. Which is given by $\Delta n^{\pm }(x,y,\delta \nu )= -\Delta \alpha ^{\pm }(x,y,\delta \nu ) \delta \nu c/2 \nu _{r}\Gamma$, where *c* is the speed of light in vacuum. Consider $n^{+}(x,y,\delta \nu )$ and $n^{-}(x,y,\delta \nu )$ are the refractive indices of $\hat{\sigma }^{+}$ and $\hat{\sigma }^{-}$ polarisation components of the imaging laser beam. For length *L* of the glass cell, the electric field of the imaging laser beam at the entrance of the glass cell is given by $E_{or}e^{-i(2\pi \nu _{r}t-k z)} \hat{y}$, where $E_{or}$ is the electric field amplitude. Therefore, the electric field of the imaging laser beam, after propagation through the atomic medium is given by

6 $$\begin{aligned} \vec {E}_{r}(x,y,\delta \nu )=E_{or}e^{-i2\pi \nu _{r}t}\left( \frac{ e^{-\alpha ^{+}(x,y,\delta \nu )L}e^{i n^{+}(x,y,\delta \nu ) kL}\hat{\sigma }^{+}-e^{-\alpha ^{-}(x,y,\delta \nu )L} e^{i n^{-}(x,y,\delta \nu )kL}\hat{\sigma }^{-}}{i\sqrt{2}}\right) \end{aligned}$$

As a consequence of different absorption and phase shift experienced by $\hat{\sigma }^{+}$ and $\hat{\sigma }^{-}$ components of polarisation, the imaging laser beam transmitted through the atomic medium becomes elliptically polarised. However, for $\alpha ^{+}(x,y,\delta \nu )\approx \alpha ^{-}(x,y,\delta \nu )$, the atomic medium becomes circularly birefringent. Therefore, the transmitted electric field of the imaging laser beam is written as

7 $$\begin{aligned} \vec {E}_{r}(x,y,\delta \nu )=E_{or}e^{-i2\pi \nu _{r}t} e^{-\alpha (x,y,\delta \nu )L}e^{i n^{+}(x,y,\delta \nu ) kL}\left( \frac{\hat{\sigma }^{+}- e^{i (n^{-}(x,y,\delta \nu )-n^{+}(x,y,\delta \nu ))kL}\hat{\sigma }^{-}}{i\sqrt{2}}\right) \end{aligned}$$

where $\alpha (x,y,\delta \nu )=\alpha ^{+}(x,y,\delta \nu )\approx \alpha ^{-}(x,y,\delta \nu )$. As a result, the transmitted imaging laser beam exhibits a position and detuning dependent rotation of its plane of polarisation. This rotation is opposite for $\phi (x,y)=\pi /2$ and $\phi (x,y)=-\pi /2$ at a given detuning $\delta \nu$ and therefore, at $p_{z}=2 m\pi \delta \nu /k$. Since this rotation depends on the transverse location on the imaging laser beam wavefront, therefore an image is formed by passing the beam through an analysing polariser P_3_, with its pass-axis oriented at an angle $-\,45^{\circ }$ *w.r.t* *x*-axis, and by detecting its transverse intensity profile by an imaging camera-1. The resulting intensity profile is given by

8 $$\begin{aligned} I_{r}(x,y,\delta \nu )=I_{or}\left( \frac{1-\sin \big (n^{+}(x,y,\delta \nu )-n^{-}(x,y,\delta \nu )\big )kL}{2}\right) \end{aligned}$$

where $I_{or}= c \epsilon _{o} |E_{or}|^{2} e^{-2\alpha (x,y,\delta \nu )L}/2$, where $\epsilon _{o}$ is the vacuum permittivity. This intensity profile represents a 2D section transverse to the $p_{z}$-axis of the 3D phase-space localised pattern. This sectional image at a detuning $\delta \nu$ is a sub-tomograph around a momentum component $p_{z}$.

## Experiment and results

An experiment is performed with gaseous ^87^Rb atoms filled in a glass cell of length $L=10$ cm, which is shielded from the external magnetic field. A uniform magnetic field $\sim 0.5$ G is applied along the *z*-axis in the glass cell. The main experimental schematic is shown in Fig. 3. Imprinting laser light is produced by a single mode extended cavity diode laser of linewidth 1 MHz. Laser frequency is locked to the $D_{2}$ transition of ^87^Rb atoms with a hyperfine ground state $|F_{g}=2\rangle$ and an excited state $|F_{e}=3 \rangle$ at $\lambda =780$ nm.

**Figure 4:**
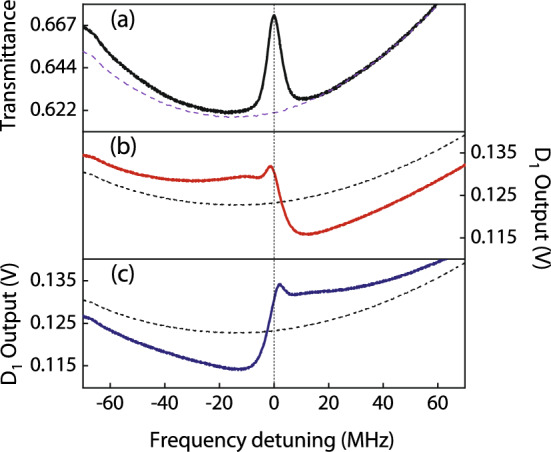
(**a**) Transmittance of the atomic medium when both laser beams are linearly polarised. (**b**) Photodetector $D_{1}$ output voltage, when the imprinting laser beam is $\hat{\sigma }^{+}$ polarised. (**c**) When the imprinting laser beam is $\hat{\sigma }^{-}$ polarised. The dotted line represents the same plot without the imprinting laser beam. The imaging laser beam is linearly polarised in all plots.

The imprinting laser light is passed through a single mode optical fibre to obtain a large diameter gaussian beam, which is collimated by a collimator. The collimated beam is retro-reflected from SLM. The retro-reflected beam is made to propagate along the $-z$-axis in the atomic medium, and it represents the imprinting laser beam. Imaging laser light is produced by a different single mode extended cavity diode laser of linewidth 1 MHz, which is locked to the same transition, but its frequency can be shifted by the acoustic-optic modulators. Imaging laser light is passed through a single mode optical fibre to obtain a large diameter collimated gaussian beam. This collimated imaging laser beam is horizontally polarised along the *y*-axis by a polariser P_2_. This polarised imaging laser beam propagating along *z*-axis is overlapped with the imprinting laser beam in the glass cell. Intensity of the imaging laser beam is $\sim \,90$ $\upmu$W/cm^2^ and imprinting laser beam is $\sim \,1.2$ mW/cm^2^. In this experiment, it is essential to measure a precise frequency difference between two laser beams. This is measured by shifting the frequency of an extracted imprinting laser light by $-\,110$ MHz and overlapping it with another extracted imaging laser light on a fast response photodetector D_2_ as shown in Fig. 3b. These two laser beams interfere in the time domain with beat frequency equals to a difference of laser frequencies. The frequency of a laser beat signal is measured by a radio frequency spectrum analyser and detuning of the imaging laser is evaluated. Rotation of a plane of polarisation of the imaging laser beam, after its propagation through the atomic medium, is analysed by a polariser P_3_. Its transverse intensity profile is measured by an EMCCD imaging camera-1 to construct a subtomographic image. A part of the imaging laser beam is reflected by BS-3 and a mirror onto a large area photodetector D_1_ after passing it through an analyzing polariser P_4_ with its pass-axis oriented at an angle $-45^{\circ }$ *w.r.t.* *x*-axis. This additional arrangement is used to obtain a frequency response of the atomic medium prior to the tomographic imaging experiment, as shown in Fig. 4. Where Fig. 4a represents the transmittance of the atomic medium when imaging and imprinting laser beams are linearly polarised and no pattern is displayed on SLM. An increase in the transmittance at the resonance is due to the saturation of absorption caused by the imprinting laser beam. However, in contrast to an experiment described in Ref.^1^ where transmittance is important to construct a tomograph, in the present experiment the transmittance variation is not critically important. The detuning of the imaging laser beam is measured *w.r.t* the peak of transmittance. In Fig. 4b, a $\hat{\sigma }^{+}$ polarised imprinting laser beam is passed through the atomic medium, which produces a rotation of the plane of polarisation of the imaging laser beam, which is measured by P_4_ and photodetector D_1_ at different detuning $\delta \nu$. In Fig. 4c, a $\hat{\sigma }^{-}$ polarised imprinting laser beam is passed through the atomic medium, which produces an opposite rotation of the plane of polarisation of the imaging laser beam. A dotted line in plots of Fig. 4a–c is the medium response, when only the imaging laser beam is passed through it and the imprinting laser beam is blocked. This experiment signifies the effect of imprinting laser beam on the atomic medium. In Fig. 4a, imaging laser power is twenty percent higher than other plots. The atomic medium shows a detectable response for different circular polarisations. After this measurement, a subtomographic imaging experiment is performed.

**Figure 5:**
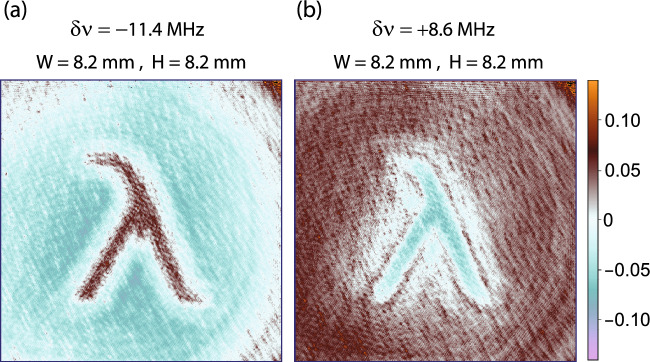
Two sub-tomographs of a 3D phase-space localised pattern. Each plot is an experimentally constructed subtomographic image, $p(x,y,\delta \nu )=-\ln (I_{r}(x,y,\delta \nu )/I_{i}(x,y,\delta \nu ))$. (**a**) For $\delta \nu =-\,11.4$ MHz. (**b**) An inverted image for $\delta \nu = +8.6$ MHz. Where the image levels of images (**a**) and (**b**) are inverted *w.r.t.* each other.

The experiment is controlled by a Lab-View program. A 3D position-space localised pattern shown in Fig. 2a is displayed on the SLM. To obtain a sub-tomograhic image of the 3D phase-space localised pattern, four different images are acquired for 200 ms time of exposure on EMCCD imaging camera-1, where each image is separated by a time interval of 700 ms from an adjacent image. The first image is discarded, and it is captured to clear noise accumulated on the EMCCD camera. A second image is captured in the presence of an imaging laser beam, in the absence of an imprinting laser beam and it is denoted by $I_{i}(x,y,\delta \nu )$. A third image is captured in the presence of imaging and imprinting laser beams. It corresponds to the image field and it is denoted by $I_{m}(x,y,\delta \nu )$. A fourth image denoted by $I_{f}(x,y)$ is captured in the absence of the imaging laser beam and in the presence of an imprinting laser beam. This image captures only the scattered light of an imprinting laser beam. A background corrected image field is given by $I_{r}(x,y,\delta \nu )=I_{m}(x,y,\delta \nu )-I_{f}(x,y)$. A final subtomographic image at a detuning $\delta \nu$ is constructed, which is given by

9 $$\begin{aligned} p(x,y,\delta \nu )=-\ln \left( \frac{I_{r}(x,y,\delta \nu )}{I_{i}(x,y,\delta \nu )}\right) \end{aligned}$$

Therefore, $p(x,y,\delta \nu )$ is positive if the plane of polarisation is rotated away from the pass-axis of polariser P_3_ and negative if the plane of polarisation is rotated towards the pass-axis, which is also evident from Fig. 4. Two experimentally constructed subtomographic 2D images are shown in Fig. 5, where (a) corresponds to a transverse section at $\delta \nu =-\,11.4$ MHz and (b) corresponds to a transverse section at $\delta \nu =+\,8.6$ MHz. The image levels of these two subtomographic images are inverted *w.r.t* each other.

Spatial resolution representing a smallest resolvable distance between two regions which differ by phase contrast $\pi$ is experimentally estimated. A one-dimensional square wave polarisation-sensitive phase pattern of phase contrast $\pi$ and fifty percent duty cycle is imprinted onto the phase-space and tomographically imaged. The period of the square wave is reduced until a tomograph becomes almost blurred. In this way, the measured spatial resolution is about 500 $\upmu$m. Spatial resolution depends on overlapping of imprinting and imaging laser beams. These two beams should be counter propagating. Diffraction of propagating imprinting laser beam and counter-propagating imaging laser beam in the medium limits the spatial resolution. The width of a pattern displayed on the SLM shown in Fig. 2a is chosen close to the estimated value of spatial resolution. In addition to spatial resolution, momentum or frequency resolution is equally important. The momentum resolution is classified in two categories, which are (1) a minimum separation between resolvable tomographs on the momentum axis and (2) a minimum separation between resolvable sub-tomographs on the momentum axis. Two different tomographs can be resolved if they are separated by about 100 MHz, which can be reduced by decreasing the intensity of the imprinting laser beam. However, two sub-tomographs of opposite contrast levels can be resolved without overlapping around the resonance with frequency resolution of about 6 MHz, where the equivalent momentum resolution is evaluated using $p_{z}=2 m\pi \delta \nu /k$.

## Conclusion

A position-space localised transparent polarisation-sensitive phase pattern is imprinted onto a unique 3D phase-space of a Doppler broadened atomic gaseous medium. This phase-space localised pattern is subtomographically imaged by an imaging laser beam around a momentum component $p_{z}=0$. Two transverse sections of the 3D phase-space localised pattern are constructed corresponding to a positive and negative detuning of the imaging laser beam. These two sectional images are inverted *w.r.t.* each other. This experiment presents a concept to imprint a position-space localised transparent polarisation-sensitive phase pattern onto a unique 3D phase-space of atoms and a subtomographic imaging of the 3D phase-space localised pattern. Different position-space transparent images can be selectively labelled by their momentum in the phase-space. Once the position-localised pattern is imprinted onto a phase-space, it becomes completely delocalised in the position and in the momentum space. Two sub-tomographs are separated by 20 MHz. These different images are extracted tomographically from the phase-space at different frequencies of the imaging laser beam. From a direct application perspective, an advantage of this type of imprinting and imaging is the extremely narrow frequency selectivity offered by the atomic medium, which corresponds to a few tens of MHz. This is an extremely narrow optical multi-band pass image filter. However, the main focus of this paper is on the foundational significance of the concept introduced in this paper and its experimental feasibility.

## Methods

Two independent extended cavity diode lasers are used to produce an imprinting laser beam and an imaging laser beam. Laser frequency can be precisely varied in steps of 1 MHz, where the linewidth of each laser is about 1 MHz. The frequency of each laser is stabilised to $D_{2}$ atomic transition $F=2$ to $F^\prime =3$ of ^87^Rb at wavelength 780 nm by using saturation absorption spectroscopy. The transverse mode of each laser output light is filtered by passing it through polarisation maintaining optical fibres. The output of an optical fibre produces a gaussian beam, which is collimated to obtain a very broad gaussian beam. In experiment, the frequency of an imprinting laser beam is not varied but the frequency of an imaging laser beam is varied. The frequency difference of both laser light beams is measured by a time domain interferometer by overlapping both laser beams with the same polarisation on a nanosecond time-response photodetector. The output of this fast-response photodetector is measured by a radio frequency spectrum analyser to monitor the frequency difference continuously. Atomic medium is a rubidium vapour cell of length 10 cm. Polarisation-sensitive phase patterns are generated by SLM, which changes the phase of the horizontal component of polarisation only. Reflectivity of SLM surface is about 0.8. The experiment is controlled by Lab-View and data is collected by an EMCCD camera-1 (Andor EMCCD camera). Four different images are taken for 200 ms exposure of camera and each image is taken after an interval of 700 ms. The first image is discarded to clear the noise accumulation when the camera was idle for a long time. The second image is taken in the absence of the imprinting laser beam, the third image is taken in the presence of both beams and the fourth image is taken in the absence of imaging laser beam. The fourth image is subtracted from the second and third images as it corresponds to a stray light field image. The required image shown in Fig. 5 is constructed from the last three images. Each image is captured twenty times in twenty repetitions of the experiment. The final image is an average of twenty repetitions of the experiment.

## Data Availability

All data generated or analysed during this study are included in this published article.
